# Microenvironment Self-Adaptive Ce-Ag-Doped Mesoporous Silica Nanomaterials (CA@MSNs) for Multidrug-Resistant Bacteria-Infected Diabetic Wound Treatment

**DOI:** 10.3390/molecules30081848

**Published:** 2025-04-20

**Authors:** Wuhao Yang, Hui Yuan, Hao Sun, Jiangshan Hu, Yaping Xu, Yuhang Li, Yan Qiu

**Affiliations:** 1 College of Materials, Xiamen University, Xiamen 361005, China; 37420222204264@stu.xmu.edu.cn; 2 State Key Laboratory of Structural Chemistry, Fujian Institute of Research on the Structure of Matter, Chinese Academy of Sciences, Fuzhou 350002, China; xmyuanhui@fjirsm.ac.cn (H.Y.); xmsunhao@fjirsm.ac.cn (H.S.); hjs5231@163.com (J.H.); 3 Xiamen Key Laboratory of Rare Earth Photoelectric Functional Materials, Xiamen Institute of Rare Earth Materials, Haixi Institutes, Chinese Academy of Sciences, Xiamen 361021, China; 4 Key Laboratory of Functional and Clinical Translational Medicine, Xiamen Medical College, Fujian Province University, Xiamen 361023, China; ypxu@xmmc.edu.cn; 5 School of Medicine, Xiamen University, Xiamen 361102, China; 6 Xiamen Key Laboratory of Chiral Drugs, Xiamen 361102, China

**Keywords:** diabetic wound treatment, reactive oxygen species (ROS), nanozyme, anti-inflammation, antibacteria

## Abstract

Chronic wound healing remains a major challenge in diabetes management due to prolonged inflammation, autonomic neuropathy, and bacterial infections. In particular, multidrug-resistant bacterial infections are important to the development of diabetic wounds, leading to persistent inflammation and delayed healing. To address this issue, we developed a self-adaptive nanozyme designed to modulate infectious and inflammatory microenvironments by doping Ce and Ag into mesoporous silicon nanomaterials (MSNs). The resulting CA@MSNs exhibited strong bacterial capture capabilities via electrostatic attraction. Additionally, the synergistic effects of Ce and Ag endowed CA@MSNs with peroxidase (POD)-like activity, enabling the generation of reactive oxygen species (ROS) to eradicate bacteria in infectious microenvironments. Notably, CA@MSNs also demonstrated the ability to scavenge a broad spectrum of ROS, including hydroxyl free radicals, hydrogen peroxide, and superoxide radicals, in inflammatory microenvironments. This dual functionality helped mitigate inflammation and promote endothelial cell migration. Consequently, treatment with CA@MSNs significantly reduced inflammation, enhanced fibroblast activation, and facilitated collagen deposition, ultimately accelerating the healing of methicillin-resistant *Staphylococcus aureus* (MRSA)-infected wounds in diabetic mice. In conclusion, this study presents a promising therapeutic strategy for chronic diabetic wounds, offering a novel approach to overcoming infection-related healing delays.

## 1. Introduction

Currently, 463 million people worldwide suffer from diabetes, a prevalent metabolic disease associated with severe complications that increase mortality rates [1]. Among these, approximately 30% of diabetic patients develop diabetic foot ulcers, which are one of the most dangerous consequences, often leading to amputation and permanent disability [1]. The pathophysiology of DFUs is closely linked to bacterial infections, impaired angiogenesis, and persistent inflammation [2]. Bacterial infections not only lower the pH of the local microenvironment but also exacerbate inflammation in diabetic wound tissues, further hindering the healing process [3]. Additionally, poor angiogenesis at wound sites induces oxidative stress, resulting in chronic inflammation and reduced collagen synthesis. For decades, antibiotics and surgical debridement have been the primary clinical strategies for managing DFUs [4]. However, excessive antibiotic use has led to the emergence of multidrug-resistant (MDR) bacteria, which makes it more challenging to treat infections with traditional treatments [5]. Therefore, novel treatment strategies that may successfully treat MDR infections while reducing inflammation and promoting angiogenesis in diabetic wounds are desperately needed.

For infections caused by multidrug-resistant (MDR) bacteria, such as methicillin-resistant *Staphylococcus aureus* (MRSA), various reactive oxygen species (ROS)-based treatments are currently under investigation [6]. ROS can effectively damage bacterial cell membranes, proteins, and nucleic acids, thereby disrupting metabolism, inhibiting growth, and even inducing cell death [7]. However, during the wound-healing phase, excessive ROS can also cause oxidative stress, severely impairing tissue regeneration [8]. Consequently, relying solely on antibacterial ROS-based treatments presents limitations in achieving optimal wound-healing outcomes. Nanozymes, including inorganic materials and metal–organic frameworks, have shown high therapeutic efficacy against MRSA infections and chronic wound healing [9]. Yao et al. developed a bimetallic BiPt nanozyme that generates multiple types of reactive oxygen species (ROS) under ultrasound stimulation, effectively combating multidrug-resistant (MDR) infections [10]. Similarly, Hu et al. designed copper–gallic acid–vancomycin nanoneedles that induce bacterial cuproptosis-like death while promoting wound healing through ROS generation [11]. While these nanozymes can generate ROS for bacterial eradication, they often lack the ability to scavenge ROS during the later stages of wound healing, potentially leading to excessive ROS accumulation and delayed tissue repair [12]. Therefore, there is a critical need to develop a microenvironment-responsive nanozyme capable of producing ROS for bacterial elimination during the acute infection phase while efficiently scavenging ROS during the subsequent wound-healing phase.

Cerium (Ce) is a therapeutic ion with unique pharmacological properties, including antibacterial and anti-inflammatory effects [13]. It exists in two oxidation states, namely Ce^4+^ and Ce^3+^. In acidic conditions (pH 4-5), typical of bacterial infections, Ce^4+^ acts as a prooxidant, generating ROS to combat pathogens [14,15,16,17]. In physiological conditions (pH 7.4), characteristic of the wound-healing phase, Ce^3+^ functions as an antioxidant, scavenging excess ROS to mitigate oxidative stress [14,15,16,17]. The catalytic efficiency of Ce is further influenced by doped metal elements, which regulate the ratio of Ce^4+^ to Ce^3+^, thereby enhancing its catalytic performance [18]. Building upon this concept, we developed a microenvironment-responsive Ce-Ag-doped mesoporous silica nanomaterial (CA@MSNs) for MRSA-infected diabetic wound treatment. Silver (Ag), a well-known antibacterial ion [19], was incorporated to enhance the ROS-scavenging capability of Ce in physiological conditions. Mesoporous silicon nanomaterials (MSNs) were employed as a substrate due to their high surface area and thermal stability, facilitating nanoparticle stabilization, preventing agglomeration, and promoting dispersion [20]. The resulting CA@MSNs exhibited dual functionality—they generated ROS in infectious microenvironments for bacterial sterilization while scavenging ROS in wound-healing microenvironments to reduce inflammation and oxidative stress. As a result, CA@MSNs not only eliminated MRSA but also promoted endothelial cell proliferation under oxidative stress conditions, thereby significantly accelerating MRSA-infected diabetic wound healing. In summary, this study highlights CA@MSNs as a promising therapeutic agent for diabetic wound healing, offering a smart, adaptive approach to combating MDR infections while promoting tissue regeneration.

## 2. Results and Discussion

### 2.1. Preparation and Characterization of CA@MSNs

**Figure 1:**
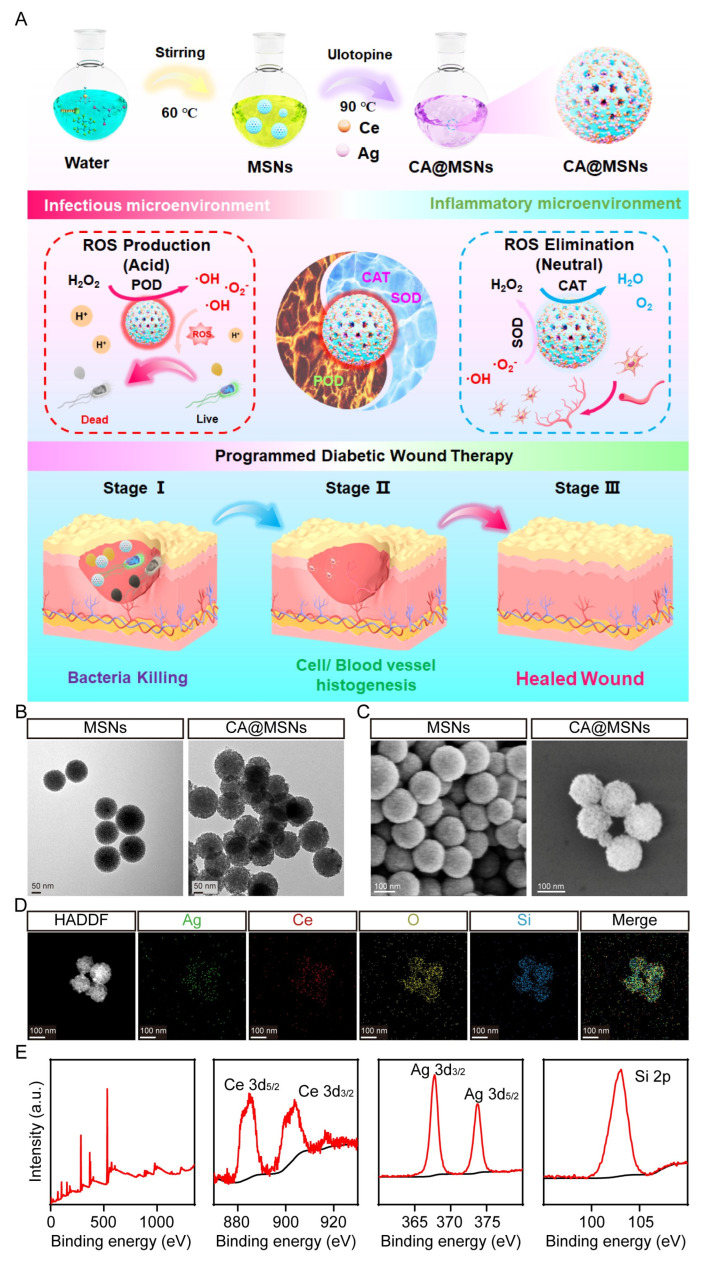
**Synthesis and characterization of CA@MSNs.** (**A**) Schematic diagram illustrating the synthesis of CA@MSNs with microenvironment self-adaptive ROS-generating and scavenging activities for treating MRSA-infected diabetic wounds in mice. (**B**) Transmission electron microscopy (TEM) and (**C**) scanning electron microscopy (SEM) images of CA@MSNs. (**D**) High-angle annular dark-field scanning transmission electron microscopy (HAADF-STEM) image of CA@MSNs, along with energy-dispersive X-ray spectroscopy (EDS) mapping images of Ce, Ag, Si, and O for CA@MSNs. (**E**) X-ray photoelectron spectroscopy (XPS) survey spectra of CA@MSNs.

Figure 1A illustrates the straightforward one-pot synthesis of Ce-Ag-doped nanoparticles (CA@MSNs). Similarly, Ce@MSNs, which contain non-doped Ce centers, were synthesized using a comparable approach. Notably, the CA@MSNs synthesis method is both simple and scalable, making it suitable for large-scale production. To characterize the structure of CA@MSNs, we employed transmission electron microscopy (TEM) and scanning electron microscopy (SEM) (Figure 1B,C). Dynamic light scattering (DLS) assay, SEM and TEM images revealed that CA@MSNs exhibited a uniform spherical shape with an average diameter of nearly 200 nm, whereas MSNs displayed a characteristic mesoporous structure with consistent pore diameters and topologies (Figure 1B,C). Furthermore, elemental mapping images confirmed the homogeneous distribution of Ce, Ag, and Si within CA@MSNs (Figure 1D). Furthermore, X-ray photoelectron spectroscopy (XPS) validated the composition of CA@MSNs, showing characteristic peaks at 368 eV and 374 eV corresponding to Ag, and peaks at 887 eV and 916 eV attributed to Ce (Figure 1E). Additionally, CA@MSNs contained approximately 2% Ce and 0.91% Ag. These findings collectively confirm the successful synthesis of CA@MSNs.

### 2.2. pH-Switchable ROS-Generating and Scavenging Activity of CA@MSNs In Vitro

**Figure 2:**
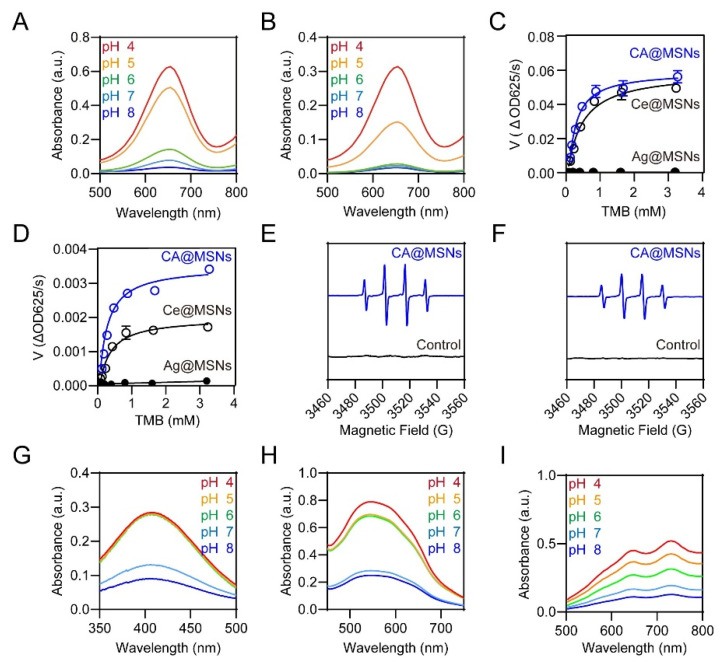
**pH-Switchable ROS-generating and scavenging activity of CA@MSNs.** (**A**) POD-like activity and (**B**) OXD-like activity of CA@MSNs under different pH conditions. Michaelis–Menten kinetic analysis for CA@MSNs in (**C**) POD-like activity and (**D**) OXD-like activity. ESR spectra of DMPO/•OH solution in the (**E**) POD-like activity test and (**F**) OXD-like activity test, with and without CA@MSNs. (**G**) CAT-like activity, (**H**) SOD-like activity, and (**I**) ABTS radical scavenging tests of CA@MSNs under different pH conditions.

The natural peroxisome is a multifunctional organelle containing peroxidase (POD), which catalyzes the breakdown of H_2_O_2_ into reactive oxygen species (ROS), and oxidase (OXD), which converts O_2_ into ROS [21]. To assess the potential of CA@MSNs as pH-switchable ROS-generating (POD-like and OXD-like) and ROS-scavenging (catalase-like, CAT) nanomaterials, we conducted a series of investigations. The POD-like and OXD-like activities of CA@MSNs were first evaluated using 3,3′,5,5′-tetramethylbenzidine (TMB) as an indicator. ROS can oxidize TMB, producing a blue-colored product with a characteristic absorption peak at 652 nm. Under acidic conditions (pH 5.0) mimicking an infectious microenvironment, CA@MSNs exhibited the highest POD-like and OXD-like activity, as indicated by the strongest absorption at 652 nm (Figure 2A,B). However, under physiological conditions (pH 7–8), CA@MSNs displayed minimal POD-like and OXD-like activity. To further quantify enzymatic performance, the maximal reaction velocity (V_m_) and Michaelis constant (K_m_) of CA@MSNs were measured at pH 5.0. Compared to non-doped Ce@MSNs (POD-like: 0.056 a.u./s, OXD-like: 0.0019 a.u./s), CA@MSNs demonstrated enhanced catalytic efficiency with higher V_m_ values (POD-like: 0.066 a.u./s, OXD-like: 0.0035 a.u./s) (Figure 2C). Additionally, the K_m_ values of CA@MSNs (POD-like: 0.22 mM, OXD-like: 0.23 mM) were lower than those of Ce@MSNs (POD-like: 0.43 mM, OXD-like: 0.29 mM), indicating a stronger substrate affinity (Figure 2D). In contrast, non-doped Ag@MSNs exhibited neither POD-like nor OXD-like activity under any conditions (Figure 2C,D). To identify the ROS species generated, we conducted an electron spin resonance (ESR) assay using 5-dimethyl-1-pyrroline N-oxide (DMPO) as a trapping agent [22]. DMPO reacts with hydroxyl radicals (•OH) to form DMPO/•OH spin adducts. As shown in Figure 2E,F, CA@MSNs significantly enhanced DMPO/•OH signals, confirming their ability to accelerate •OH production via O_2_ (OXD-like activity) and H_2_O_2_ (POD-like activity). Together, these findings demonstrate that CA@MSNs exhibit excellent ROS-generating capabilities in infectious microenvironments, functioning similarly to POD and OXD enzymes.

Next, we investigated the ability of CA@MSNs to mimic catalase (CAT)-like activity by reducing H_2_O_2_ to O_2_ and superoxide dismutase (SOD)-like activity by converting superoxide anions (O_2_•^−^) into H_2_O_2_ and O_2_. As expected, CA@MSNs exhibited pH-dependent CAT-like and SOD-like activity (Figure 2G,H). Under physiological conditions (pH 7–8), CA@MSNs demonstrated strong CAT-like and SOD-like activity, efficiently catalyzing the decomposition of ROS. However, in acidic infectious microenvironments (pH 5–6), their activity was significantly diminished. Additionally, we assessed the ROS-scavenging capability of CA@MSNs using the ABTS assay. In this test, the reaction between ABTS and potassium persulfate generates the reactive radical cation ABTS^+^•. The antioxidant potential of CA@MSNs was confirmed by the dose-dependent reduction in ABTS^+^• absorption in phosphate-buffered saline (PBS), demonstrating their strong ROS-scavenging properties under physiological conditions (Figure 2I).

### 2.3. Antibacterial Activities of CA@MSNs on MDR

**Figure 3:**
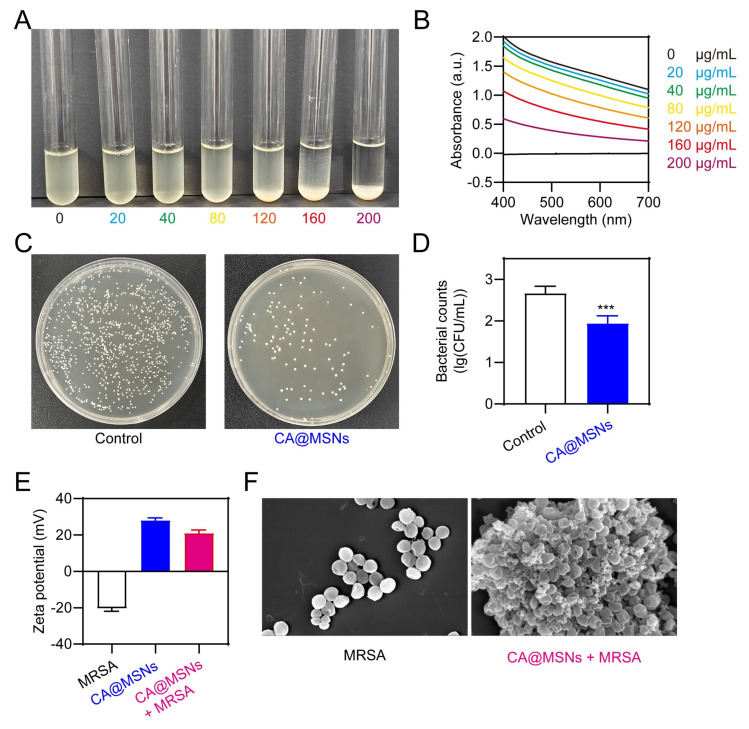
**Antibacterial activities of CA@MSNs against MRSA.** (**A**) Photographs and (**B**) absorbance at 600 nm of MRSA incubated with various concentrations of CA@MSNs. (**C**) Photographs of agar plates and (**D**) corresponding bacterial colony counts following CA@MSN treatment, ***, *p* < 0.001 vs. Control group. (**E**) The zeta potential profile of CA@MSNs, MRSA, and their mixture in aqueous solutions. (**F**) SEM images of MRSA treated with or without CA@MSNs.

We assessed the UV–visible absorbance of bacterial suspensions after treatment and performed a growth-inhibition experiment in bacterial cultures to further assess the antibacterial capabilities of CA@MSNs. Since bacterial turbidity correlates with absorption at 600 nm, this measurement served as an indicator of bacterial density [23]. Initially, we examined the bacterial suspensions’ and CA@MSNs’ UV–visible absorption spectra. While the CA@MSNs solution exhibited mild UV–visible absorption, the MRSA suspension displayed a strong absorption peak at 600 nm (Figure 3A,B). Notably, mixing CA@MSNs with the MRSA solution did not alter the bacterial suspension’s absorption spectrum, indicating that CA@MSNs did not directly interfere with UV–visible absorption (Figure 3A,B). The antibacterial activity of CA@MSNs was demonstrated by a dose-dependent reduction in the UV absorbance of the MRSA culture medium following treatment (Figure 3A,B). We employed the traditional plate count method to further confirm their bactericidal efficacy. As shown in Figure 3C,D, CA@MSNs exhibited potent antibacterial activity, significantly reducing MRSA colony formation. Additionally, MRSA showed a negative surface charge with a zeta potential of −20.43 mV, whereas CA@MSNs showed a positively charged surface with a zeta potential of +28.03 mV (Figure 3E). When CA@MSNs were added to the MRSA suspension, the zeta potential showed a notable shift, going from −20.43 mV to +21.02 mV. This indicates a strong interaction between the two components. SEM was used to analyze the morphological changes in MRSA cells following CA@MSN treatment. In the untreated group, bacterial cells displayed smooth and intact membranes. In contrast, CA@MSNs-treated MRSA exhibited rough, sunken, and structurally deformed membranes (Figure 3F), indicating severe membrane disruption. Collectively, these results confirm that CA@MSNs possess strong antibacterial activity against antibiotic-resistant MRSA by compromising bacterial membrane integrity.

### 2.4. ROS-Scavenging and Angiogenic Activity of CA@MSNs in HUVECs

**Figure 4:**
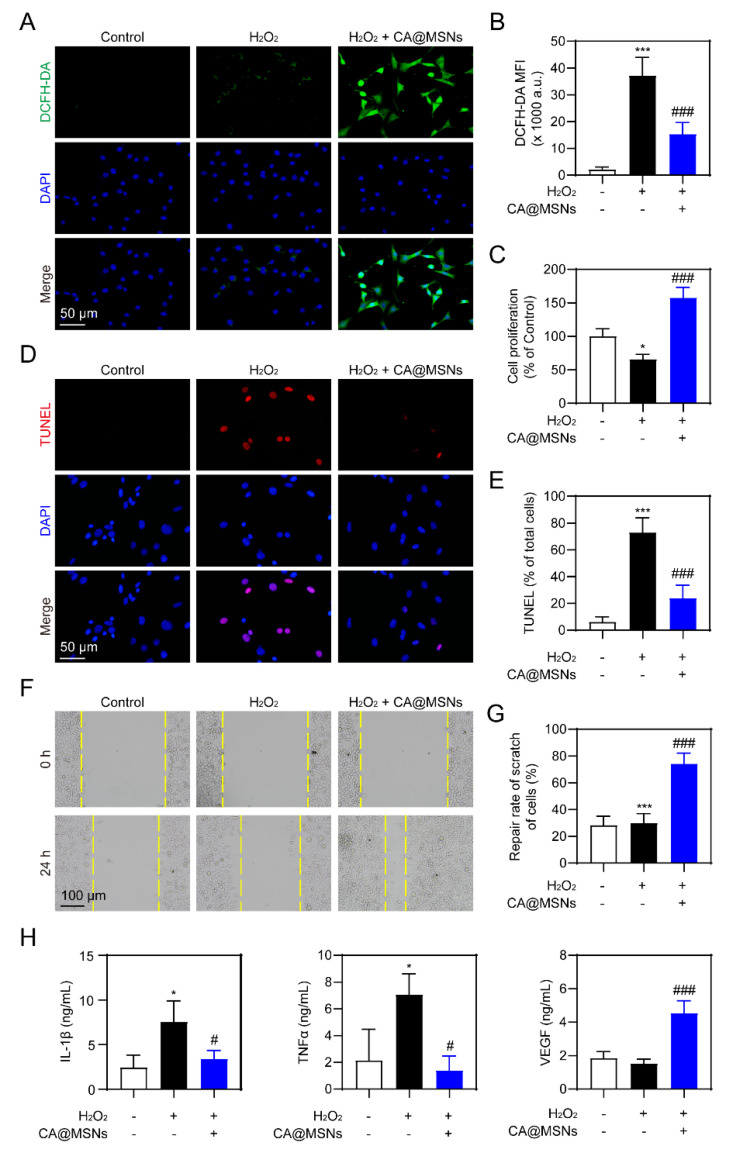
**ROS-scavenging and angiogenic activity of CA@MSNs in HUVECs.** (**A**) Representative confocal images and (**B**) quantification of ROS levels in HUVECs. (**C**) CCK-8 assay of HUVEC viability with or without CA@MSNs. (**D**) Representative confocal images and (**E**) quantification of TUNEL-positive HUVECs. (**F**,**G**) Cell migration assay (scratch assay) showing the effect of CA@MSNs. (**H**) ELISA quantification of IL-1β, TNF-α, and VEGF levels in HUVEC culture supernatants. * *p* < 0.05, *** *p* < 0.01 vs. Control group; # *p* < 0.05, ### *p* < 0.01 vs. H_2_O_2_ + vehicle group.

Endothelial cells play a crucial role in angiogenesis, a key process in wound healing. However, oxidative stress in diabetic wounds impairs endothelial cell migration and survival, significantly hindering the healing process [24]. To evaluate whether ROS scavenging by CA@MSNs could enhance cell survival under oxidative stress, we incubated human umbilical vein endothelial cells (HUVECs) in a complete culture medium containing 10 µM H_2_O_2_ and treated them with CA@MSNs. A CCK-8 assay showed that CA@MSNs (100 µg/mL) showed low cytotoxicity on HUVECs (Figure S2). CA@MSN treatment markedly reduced H_2_O_2_-induced ROS generation in HUVECs, as shown in Figure 4A,B. We then assessed cell viability using the CCK8 assay, which revealed a significant decrease in HUVEC proliferation under oxidative stress compared to control groups (Figure 4C). However, CA@MSN treatment effectively mitigated this reduction, enhancing HUVEC proliferation by scavenging excess ROS (Figure 4A–C). Similarly, oxidative stress led to increased HUVEC apoptosis, but CA@MSN treatment significantly improved cell survival (Figure 4D,E). Since endothelial cell migration is also essential for wound healing, we next examined the effect of CA@MSNs on HUVEC migration using a scratch assay. After 24 h of incubation under oxidative stress conditions, HUVEC migration was noticeably reduced (Figure 4F,G). However, CA@MSN treatment successfully restored migratory capacity, stabilizing HUVEC movement (Figure 4F,G). Poor angiogenesis in diabetes can lead to prolonged, uncontrolled inflammation driven by hyperglycemia [24]. To investigate the impact of CA@MSNs on inflammatory responses in HUVECs, we performed an ELISA assay. The results showed that CA@MSNs-mediated ROS scavenging significantly reduced the levels of IL-1β and TNF-α, two key proinflammatory cytokines elevated under prolonged high-glucose exposure (Figure 4H). Furthermore, CA@MSN treatment increased the expression of VEGF, a critical growth factor involved in angiogenesis, under oxidative stress conditions (Figure 4H). Collectively, these findings indicate that CA@MSNs promote endothelial cell survival, migration, and angiogenesis while reducing oxidative stress and inflammation, making them a promising therapeutic agent for diabetic wound healing.

### 2.5. CA@MSNs Promoted MRSA-Infected Diabetic Wound Healing In Vivo

**Figure 5:**
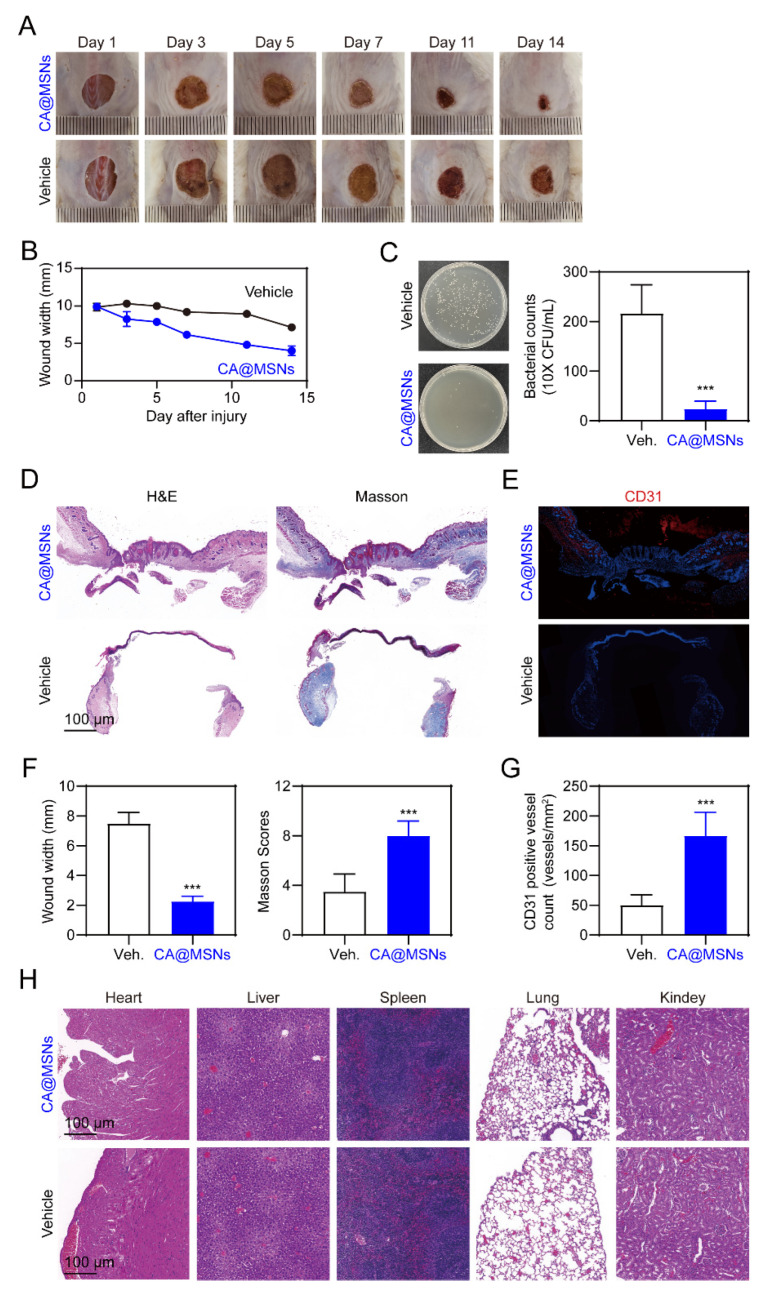
**CA@MSNs promote MRSA-infected diabetic wound healing in mice.** (**A**) Representative images showing the change in wound size on different days. (**B**) The width of the wounds treated with or without CA@MSNs on different days. (**C**) Representative images showing the number of surviving bacterial colonies on day 14 in the wound area. (**D**) H&E and Masson’s trichrome staining of the wound tissue on day 14. (**E**) Representative CD31 immunostaining images showing endothelial cell marker expression on day 14. (**F**) The width and the Masson score of the wounds treated with or without CA@MSNs on day 14. (**G**) The number of CD31-positive cells in (**E**). (**H**) H&E staining of the heart, liver, spleen, lungs, and kidneys after different treatments on day 14. ***, *p* < 0.001 vs. vehicle group.

Finally, we studied the effects of CA@MSNs on MRSA-infected wounds in mice with diabetes. Full-thickness MRSA-infected wounds were created on the dorsum of diabetic mice, and CA@MSNs were topically applied for 14 days. Gross examination of wound closure revealed that CA@MSNs significantly accelerated wound healing compared to the PBS-treated group, which exhibited no improvement (Figure 5A,B). To assess bacterial clearance, wound tissues were collected on day 14, and bacterial suspensions were plated on agar to quantify residual colonies (Figure 5C). The PBS-treated group exhibited a high number of surviving bacteria, whereas the CA@MSNs-treated group showed a significant reduction in bacterial colonies, demonstrating the potent bactericidal activity of CA@MSNs. Histopathological analysis of wound healing was performed using H&E staining. By day 14, wounds in the CA@MSNs-treated group exhibited a thicker neo-epidermis, abundant granulation tissue, reduced scar formation, and a narrower wound gap compared to the PBS-treated group (Figure 5D,F). Masson’s trichrome staining was used to further examine collagen deposition, which is an important factor in wound healing. On day 14, wounds treated with CA@MSNs displayed a well-developed dermal layer with robust collagen fiber deposition, whereas PBS-treated group wounds exhibited weaker collagen formation (Figure 5D,F). To examine angiogenesis, we assessed CD31 expression, an endogenous angiogenic marker primarily expressed by endothelial and immune cells. Immunohistochemical analysis revealed significantly higher CD31 levels in CA@MSNs-treated wounds compared to the PBS-treated group, indicating enhanced vascularization (Figure 5E,G). Furthermore, CA@MSNs-treated mice exhibited normal morphology and no visible signs of systemic toxicity, suggesting excellent biocompatibility and in vivo safety (Figure 5H). Collectively, these findings demonstrate that CA@MSNs promote bacterial clearance, accelerate wound healing, enhance collagen deposition, and stimulate angiogenesis, making them a promising therapeutic strategy for MRSA-infected diabetic wounds.

## 3. Experimental Section

### 3.1. Materials

Sinopharm (Shanghai, China) supplied all of the reagents utilized in this investigation; unless otherwise noted, the best grade commercially available was sought. Every chemical was used just as it was delivered, requiring no additional purification.

### 3.2. Synthesis of CA@MSNs

MSNs were synthesized via a one-pot method [20]. In a 60 mL solution of deionized water, 1.5 g of hexadecyltrimethylammonium bromide (CTAB) and 5 M triethylamine (Et_3_N) were combined. Then, 16 mL of tetraethyl orthosilicate (TEOS) in cyclohexane (4.7 M) was added dropwise. After being stirred at 60 °C for two days, the solution was then centrifuged for five minutes at 8000 rpm, and the supernatant—which included impurities and unreacted reagents—was disposed of. After washing the pellet with 5 × 10 mL of ethanol to remove any leftover residue, it was resuspended in 20 mL of ethanol.

To prepare the CA@MSNs, 2 mL of the MSN solution was added dropwise to 60 mL of deionized water containing 0.1 g of silver nitrate (AgNO_3_) and 0.1 g of cerium chloride (CeCl_2_). After stirring for 35 min at 90 °C, 0.1 g of urotropine in 5 mL of deionized water was added. The solution was stirred at the same temperature for two hours. The obtained CA@MSNs, loaded with Ag (0.91 wt%) and Ce (2 wt%), were centrifuged at 2000× *g* for 30 min. The product was then dried at 60 °C for 12 h.

### 3.3. Characterization

The shape and particle size of the MSNs and CA@MSNs were analyzed by SEM and TEM by utilizing field emission high-resolution transmission electron microscopy (JEOL, Peabody, MA, USA). The chemical surface species and the concentrations of Ag and Ce of CA@MSNs were investigated by XPS. The optical diffuse reflectance and UV spectra were recorded using an Agilent Cary 5000 UV–Vis–NIR spectrophotometer (Agilent, Santa Clara, CA, USA).

POD-**like activity assay**

The POD-like activity of CA@MSNs was evaluated using a TMB oxidation assay [25]. A NaOAc-HOAc buffer (1 mL, pH 5.0) contained CS@MSNs (0.1 mg/mL), TMB (800 μM) and H_2_O_2_ (0.01 mL) was incubated at room temperature for 1.5 h. By monitoring the variations in absorbance of the oxidized TMB at 652 nm, TMB oxidation was tracked.

OXD-**like activity assay**

Colorimetric assays were also employed to assess the oxidase (OXD)-like activity [26]. CA@MSNs (100 μg/mL) and TMB (800 μM) were incubated in a NaOAc-HOAc solution (1 mL, 95% O_2_, pH 5.0) at 37 °C for 30 min. By monitoring the changes in absorbance of the oxidized TMB at 652 nm, the oxidation of TMB was quantified.

DMPO **assay**

To prepare the DMPO/•OH solution, 40 µL of deionized water was mixed with 0.25 µmol of DMPO, 0.1 µmol of H_2_O_2_, and 0.1 µmol of FeCl_2_ for 5 min. To investigate the scavenging activity of CA@MSNs on •OH, ESR spectra were acquired following a two-minute incubation of 100 μg of CA@MSNs with 40 μL of DMPO/•OH solution [27].

### 3.4. Superoxide Dismutase (SOD)-like Activity Assay

The nicotinamide adenine dinucleotide (NADH)–phenazine methosulfate (mPMS)–nitroblue tetrazolium (NBT) technique was used to assess the SOD-like activity of CA@MSNs [28]. NBT can be oxidized by O_2_•^−^, formed through the reaction between NADH and mPMS, and displays a distinctive UV absorption peak at 560 nm. In this assay, NBT (5.8 µmol) in deionized water (100 µL) was added to a mixture of NADH (0.019 mmol), mPMS (6.8 µmol), and CA@MSNs (100 μg/mL) in PBS (900 µL) at varying pH levels. The residual oxidative form of NBT at 680 nm was measured by recording the UV–Vis spectra of the resulting mixture after a five-minute incubation.

### 3.5. CAT-like Activity Assay

The catalase (CAT)-like activity was evaluated using a colorimetric method involving titanium sulfate [29]. CA@MSNs (100 μg/mL) and H_2_O_2_ (3 mM) were combined in PBS (10 mL) at various pH levels and incubated for 30 min. Subsequently, a solution of Ti(SO_4_)_2_ (1 mmol) and H_2_SO_4_ (4 mL) was added to 16 mL of deionized water, and the mixture was agitated for 2 min. The reduction of H_2_O_2_ was monitored by analyzing the absorbance fluctuations of the resulting peroxotitanic acid using a UV–Vis spectrometer (Agilent, Santa Clara, CA, USA).

### 3.6. ABTS Assay

The ABTS• radical was generated by oxidizing ABTS with K_2_S_2_O_8_. ABTS (37.4 mg) was mixed with NaOAc buffer (20 mM, 30 mL) and 7 mg of K_2_S_2_O_8_ to generate the ABTS• solution, which was then agitated for 10 h at 4 °C in the dark. After a two-minute incubation time at 37 °C with CA@MSNs (100 μg/mL) in the ABTS• solution (3 mL), UV–Vis spectra were collected to test the antioxidant properties of CA@MSNs. The ABTS• radical’s absorption at 734 nm was utilized to calculate its reduction [30].

### 3.7. Binding Assay

The CA@MSNs’ zeta-average hydrodynamic diameter was assessed using the ZetaSizer Nano ZS90 (Malvern, Malvern, UK). CA@MSNs (200 µg/mL, 0.2 mL) were treated with bacterial suspensions (1 × 10^8^ CFU/mL, 0.2 mL) for two hours at 37 °C in order to evaluate the interaction between the two. Following incubation, the mixture was centrifuged at 6500× rpm for 5 min. After three rounds of washing, the resultant pellets were reconstituted in three milliliters of deionized water. The pellets were preserved in a 4% paraformaldehyde solution at 4 °C for an entire night in order to study the morphology of the CA@MSNs–bacteria complex. The materials were then progressively dehydrated using a succession of ethanol solutions with concentrations ranging from 30% to 100%. Following dehydration, the bacterial samples were collected for further examination by centrifuging them for eight minutes at 6500 rpm. For SEM, dried bacteria were used.

### 3.8. Growth-Inhibition Assay in Liquid Medium

Different concentrations of CA@MSNs (0, 40, 80, 120, 160, and 200 µg/mL) were added to bacterial suspensions of methicillin-resistant *Staphylococcus aureus* (MRSA, ATCC, Cat. #700698) in LB liquid medium (1 × 10^8^ CFU/mL, 5 mL). To assess the suppression of bacterial growth, the mixture was incubated at 37 °C for four hours. The UV absorbance at 600 nm was further measured. Each concentration was tested in triplicate [25].

### 3.9. Plate Counting

To make the solid medium, 5 g of tryptone, 5 g of sodium chloride, 2.5 g of yeast extract, and 3.75 g of agar powder were added to 500 mL of water and mixed for 30 min at 80 °C. After the mixture was transferred into 5 cm Petri dishes and allowed to firm at room temperature, the plates were covered and kept at 5 °C in the refrigerator. A 100 µL aliquot of the diluted bacterial suspension—10,000 times diluted with regular LB liquid media—was plated onto the solid medium in order to count the plates. After that, at 37 °C, the plates were incubated for 1 day. On the agar plates, the number of bacterial colonies (CFU/mL) was measured. For every treatment, a minimum of three replicates were employed [25].

### 3.10. Cells Experiments

HUVECs (Cat. # IM-H205, Immocell Biotechnology) were cultured in Ham’s F12K medium (Gibco, Cat. #21127022) supplemented with 10% fetal bovine serum (FBS) and 25 mM glucose. The cells were treated for 30 min with PBS or CA@MSNs (100 µg/mL), followed by stimulation with either water (control) or H_2_O_2_ (25 µM). After 24 h of incubation, both cells and supernatants were collected for subsequent analysis. At least three replicates were used for each treatment group. Cell viability was assessed using the CCK-8 kit (Beyotime, Cat. #C0038) following the manufacturer’s instructions

### 3.11. Enzyme-Linked Immunosorbent Assay (ELISA)

The cytokine levels in the HUVEC culture supernatants were measured using corresponding ELISA kits in accordance with the manufacturer’s instructions. VEGF (Fine Biotech, Cat. # EH0327), IL-1β (NeoBioscience, Cat. # EMC004), and TNF-α (NeoBioscience, Cat. # EMC102a) levels in supernatants were measured using ELISA kits.

### 3.12. TdT-Mediated dUTP Nick End Labeling (TUNEL) Assay

In 35 mm glass-bottomed dishes, HUVECs were seeded and fixed for 30 min in 4% paraformaldehyde. The cells were then incubated with the TUNEL reaction mixture (Promega, Cat. #G7131) for one hour at 37 °C. Afterward, they were incubated for 30 min with DAPI. Images were captured using confocal microscopy, and ImageJ software (version 1.45) was used for quantification.

### 3.13. Cell Scratch Experiment

To create a cell monolayer, HUVECs (1 × 10^6^ cells/mL) were planted in 6-well culture plates for 48 h. After using a 10 μL pipette to scratch the cell monolayer, it was incubated with PBS and CA@MSNs (100 μg/mL) for 30 min. It was subsequently treated with H_2_O_2_ (25 µM) or water (control). An inverted fluorescent microscope was used to take pictures of the cells after they had been incubated for 24 h. (S_0_ − S_24_)/S_0_ × 100% is the scratch mending rate (%), where S_0_ and S_24_ stand for the scratch areas prior to and following intervention, respectively. For every treatment, a minimum of three replicates were examined.

### 3.14. Animal

Animal experiments were carried out in compliance with ARRIVE and the National Institutes of Health’s (NIH) Guide for the Care and Use of Laboratory Animals. They also received approval by the Animal Care and Use Committee of Xiamen University.

Male BALB/c mice (8–10 week) were given streptozotocin (80 mg/kg, dissolved in 100 µL of citrate buffer, pH = 4.2–4.5) intraperitoneally (i.p.) once every day for five days in a row. The dosage of CA@MSNs was determined through preliminary experiments. Different doses are tested to evaluate both the effectiveness and safety of the drugs. Various factors, such as pharmacokinetics and desired therapeutic effects, are taken into account to establish the final dosage. Mice were deemed diabetic after four weeks if their fasting blood glucose levels were higher than 16.6 mM. Following 3% isoflurane anesthesia and hair removal, a round skin biopsy punch was used to create a full-thickness skin wound on each mouse’s back measuring 10 mm in diameter. The exposed wounds were then injected with 100 μL of 1 × 10^8^ CFU mL^−1^ MRSA bacteriophage solution. At 1, 3, 5, and 7 days following surgery, the wound was topically treated with CA@MSNs (10 mg/kg) or its Vaseline vehicle. At one, five, and fourteen days after surgery, wound healing was noted and documented, and the Image J program was used to measure and examine the wound regions.

### 3.15. Histological Analysis

Before being embedded in paraffin, skin samples from the diabetic mice’s dorsal region were gathered and stored in a 2% paraformaldehyde solution. Sections 5 µm thick were obtained using a microtome, and for histological investigation, they were stained with Masson’s trichrome and standard hematoxylin and eosin (H&E). The photos were taken using an Olympus microscope. The epithelial layer’s thickness was measured in more than a dozen different areas. A minimum of two scientists independently and blindly assessed each histology sample.

### 3.16. Immunofluorescent Staining

Mice paraffin sections were subjected to immunofluorescent examination. The immunofluorescence analyses were performed using the usual procedure. After deparaffinization and antigen retrieval, the slides were incubated with primary antibodies, specifically anti-CD31 (Proteintech, Cat. # 11265-1-AP, dilution 1:300), for an entire night at 4 °C. The sections were incubated for one hour at room temperature before being washed with 0.1 M PBS and subjected to goat anti-rabbit IgG-Alexa Fluor 488 (Abcam, Cat. ab150077, dilution 1:1000). Cell nuclei were labeled using DAPI, a counterstain that was acquired from Vector Lab in Shanghai, China. Confocal microscopy was used to detect the fluorescence and Image J software was used to quantify it.

### 3.17. Data and Statistical Analysis

The mean ± SEM was used to present the data. To conduct all statistical analyses, GraphPad Prism version 9.0.0 was used. Dunnett’s post hoc multiple comparison tests were used in conjunction with a one-way ANOVA to evaluate three or more distinct groups. Only when F reached *p* < 0.05 and there was no significant variance inhomogeneity were post hoc tests performed for all ANOVAs. The threshold for statistical significance was set at *p* < 0.05.

## 4. Conclusions

The development and progression of diabetic wounds are significantly exacerbated by multidrug-resistant (MDR) infections, which lead to chronic inflammation and delayed healing. Here, we developed the self-adaptive nanozyme CA@MSNs, designed to target both infectious and inflammatory microenvironments. CA@MSNs demonstrated potent antibacterial activity, effectively killing MRSA both in vitro and in vivo. This is attributed to their strong bacterial capture capacity and peroxidase (POD)-like ROS-producing activity in infectious environments. Additionally, in inflammatory environments, CA@MSNs efficiently scavenged a broad range of ROS, including •OH, O_2_•^−^, and H_2_O_2_, which reduced inflammation and promoted endothelial cell migration. Consequently, CA@MSNs significantly promoted the healing of MRSA-infected wounds in diabetic mice.

The findings of this study suggest that microenvironment-responsive nanomaterials, like CA@MSNs, hold considerable promise for clinical applications, particularly in mitigating oxidative stress, eliminating bacterial infections, and accelerating diabetic wound healing. This innovative approach offers a novel and effective treatment option for diabetic patients, potentially improving their quality of life and reducing the risk of complications. However, despite their promising performance and potential applications demonstrated in this study, there are still challenges in the clinical adoption of CA@MSNs. Further research is necessary to assess their long-term safety and effectiveness, particularly in addressing the various types and severities of diabetic wounds. In conclusion, this study paves the way for a promising therapeutic approach to treating chronic diabetic wounds.

## Data Availability

All data are available from the corresponding author upon reasonable request.

## Supplementary Materials

The following supporting information can be downloaded at: https://www.mdpi.com/article/10.3390/molecules30081848/s1, Figure S1. DLS assay of CA@MSNs; Figure S2. The cytotoxicity of CA@MSNs in HUVECs. N = 3.
