# Supplementary material for: Microenvironment Self-Adaptive Ce-Ag-Doped Mesoporous Silica Nanomaterials (CA@MSNs) for Multidrug-Resistant Bacteria-Infected Diabetic Wound Treatment
Source: Molecules. 2025 Apr 20;30(8):1848. doi: 10.3390/molecules30081848 (PMC12029609; doi:10.3390/molecules30081848)
Supplement: Supplementary file 1 [file molecules-30-01848-s001.zip › molecules-3562874-supplementary.pdf]

Microenvironment self-adaptive Ce-Ag doped mesoporous silica nanomaterials (CA@MSNs) for multidrug-resistant bacteria-infected diabetic wound treatment

Wuhao Yang [1], Hui Yuan [2, 3], Hao Sun [2, 3], Jiangshan Hu [2, 3], Yaping Xu [4], Yuhang Li [2, 3, 4 *], Yan Qiu [5, 6 *]

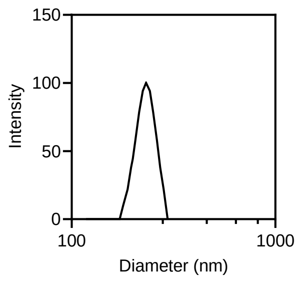


Figure S1. DLS assay of CA@MSNs

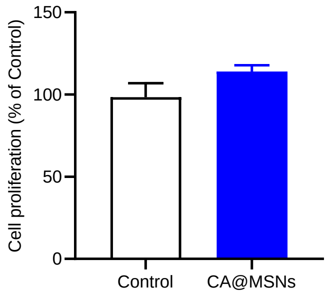


Figure S2. The cytotoxicity of CA@MSNs in HUVECs. N = 3
